# Supplementary material for: The interactome of intact mitochondria by cross-linking mass spectrometry provides evidence for coexisting respiratory supercomplexes
Source: Mol Cell Proteomics. 2017 Dec 8;17(2):216–32. doi: 10.1074/mcp.RA117.000470 (PMC5795388; doi:10.1074/mcp.RA117.000470)
Supplement: Supplemental Data [file supp_RA117.000470_133922_0_supp_23978_fzffwf.zip › spectra_annotation/mito_DR_spectra_annotation/139-1-1-1-19-1.pdf]

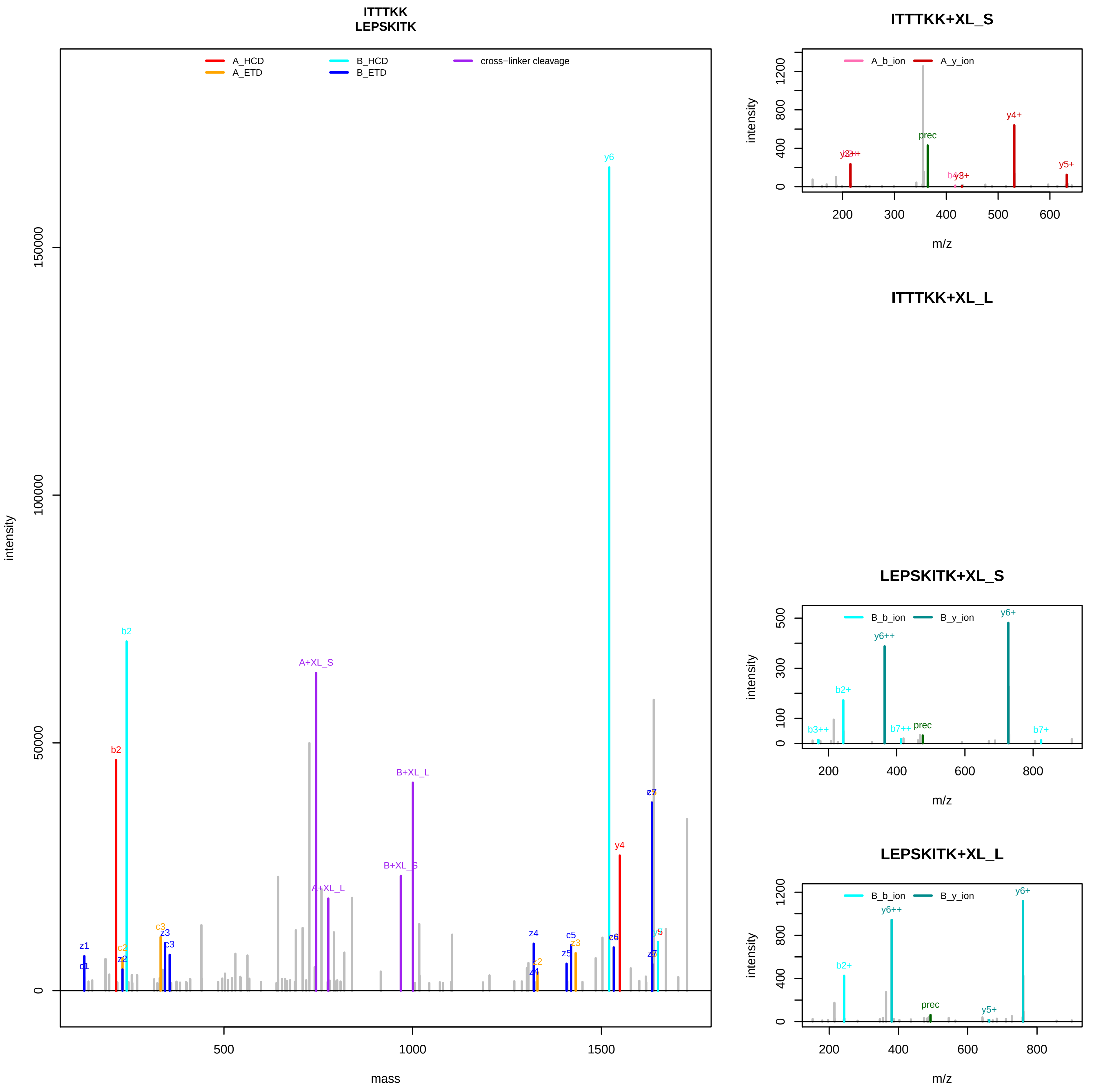

ITTTKK
LEPSKITK
A_HCD
A_ETD
B_HCD
B_ETD
cross−linker cleavage
intensity
mass
0
50000
100000
150000
500
1000
1500
y6
b2
A+XL_S
B+XL_L
B+XL_S
A+XL_L
y4
z1
c1
c2
z2
c3
z3
z4
c5
z5
c6
z7
c7
y5
ITTTKK+XL_S
A_b_ion
A_y_ion
y3++
prec
y4+
y5+
y3+
m/z
ITTTKK+XL_L
LEPSKITK+XL_S
B_b_ion
B_y_ion
y6+
y6++
b2+
b3++
b7++
b7+
LEPSKITK+XL_L
y5+
